# Targeted therapy for Langerhans cell histiocytosis with maxillofacial involvement in 20 children

**DOI:** 10.1186/s13023-026-04301-w

**Published:** 2026-03-26

**Authors:** Yang Jiang, Zhigang Li, Ying Yang, Rui Zhang, Tianyou Wang, Guoxia Yu

**Affiliations:** 1 Department of Stomatology, Beijing Children’s Hospital, Capital Medical University, National Center for Children’s Health (NCCH), Beijing, 100045 China; 2 Laboratory of Hematologic Diseases, Hematology Center, Beijing Children’s Hospital, Beijing Pediatric Research Institute, Capital Medical University, National Center for Children’s Health, Beijing, 100045 China; 3 Beijing Key Laboratory of Pediatric Hematology Oncology, Beijing Children’s Hospital, Capital Medical University, National Center for Children’s Health, Beijing, 100045 China; 4 National Key Discipline of Pediatrics, Capital Medical University, Beijing, 100045 China; 5 Key Laboratory of Major Diseases in Children, Ministry of Education, Beijing, 100045 China

**Keywords:** Langerhans cell histiocytosis, BRAF mutation, *MAP2K1* mutation, Maxillofacial manifestations, Target therapy

## Abstract

**Background:**

To evaluate targeted therapy for Langerhans cell histiocytosis (LCH) with maxillofacial manifestations, we conducted a retrospective study of 20 children admitted from January 2016 to June 2021.

**Methods:**

The maxillofacial LCH exhibited diverse clinical features and laboratory indices. Seventeen patients were refractory to standard treatments, and three patients directly underwent targeted therapy. Sixteen patients with *BRAF*^*V600E*^ mutation were treated with dabrafenib, and four patients with *MAP2K1* mutation were treated with trametinib.

**Results:**

The cohort included nine males and eleven females, with a median 0.92 years at diagnosis and 1.70 years at initiation of targeted therapy. Three patients had single-system LCH and 17 patients had multi-system LCH. Lesions affected the mandibular bone in 17 patients, the temporal bone in 12 patients, and the maxilla in 10 patients. Ki-67 expression before targeted therapy correlates with maxillary involvement (coefficient 0.61; *P* < 0.05). The *cfBRAF*^*V600E*^ level after first month of targeted therapy was significantly lower than before targeted therapy (*P* < 0.001). After a median 18 months of targeted therapy and 22 months of follow-up, 17 patients achieved complete remission, and 3 patients achieved partial remission. The objective response rate was 65%, and the disease control rate was 100%. Five patients experienced progression or reactivation, and ten patients had skin rashes.

**Conclusion:**

Overall, targeted therapy demonstrated good efficacy with mild tolerable adverse events.

**Supplementary Information:**

The online version contains supplementary material available at 10.1186/s13023-026-04301-w.

## Introduction

Langerhans cell histiocytosis (LCH) is a rare myeloid neoplastic disorder, characterized by inflammatory lesions from accumulation of CD1a+/CD207 + histiocytes [1, 2]. Maxillofacial LCH is of particular interest due to its complex clinical presentation, diagnostic challenges, and significant implications for affected children [3]. The maxillofacial region is one of the most frequently affected sites in pediatric LCH, and oral manifestations are often the first or only signs of the LCH disease [4]. The lesion can spread from the dento-alveolar region to adjacent craniofacial bones. Its clinical presentation also varies widely, ranging from solitary lesions that may regress spontaneously to multisystem disease with rapid progression even to death [5, 6]. In fact, the LCH with the maxillofacial involvement has diverse and non-specific clinical manifestations, such as masses, gingival ulcerations/lesions, gingival bleeding, oral pain or swelling, and tooth mobility or loss [7]. Since the maxilla, oral cavity, orbit, mastoid, sphenoid, zygomatic bones, or temporal bones are classified as “central nervous system (CNS) risk” [8], maxillofacial LCHs involving these sites are likely to develop the neurodegenerative CNS disease with a high risk of permanent CNS sequelae [9].

Precision diagnosis of maxillofacial LCH is challenging as maxillofacial LCH frequently mimics common pediatric dental conditions, leading to potential misdiagnosis or delayed diagnosis [10, 11]. It also requires biopsy confirmation of lesional tissues and comprehensive staging to distinguish single system from multisystem/CNS high-risk disease [12]. So, early and comprehensive recognition is critical to avoid misdiagnosis and prevent progression to multisystem disease or permanent dental/facial sequelae [13, 14].

Standard treatments for LCH include observation, bone curettage or biopsy, and first-line chemotherapy [15, 16]. However, the LCH involving the hematopoietic system, spleen, and/or the liver can be difficult to treat [17]. In addition, pediatric patients with LCH treated with aggressive and toxic salvage treatment approaches generally have poor prognosis [18]. Recent studies in molecular research have elucidated that activation of the mitogen-activated protein kinase (*MAPK*) pathway is due to gene mutations in all the LCH samples. V-Raf murine sarcoma viral homolog B1 (*BRAF*) and mitogen-activated protein kinase 1 (*MAP2K1*, also called *MEK1*) are the most frequently mutated genes, accounting for 57.0% and 20.6% of LCH patients, respectively [19]. And the *BRAF*^*V600E*^ mutation has been linked with increased resistance to first-line chemotherapy and poor prognosis in LCH [17].

Some early studies from targeted therapy have shown encouraging clinical responses in LCH patients with *BRAF*^*V600E*^ / *MAP2K1* mutations [20–23]. The completed clinical trial also evaluated the efficacy of a combination of dabrafenib and trametinib (https://clinicaltrials.gov/study/NCT02124772; NCT02124772 was registered in 2014 with study start to end date of 2015 to 2020) [23]. National comprehensive cancer network lists the guidelines for adult and pediatric LCH [24].

In short, a pre-defined sub-analysis for the maxillofacial LCH in the context of typical LCH is important, but its targeted therapy is scarcely studied [25–29], especially concerning the pediatric patients [30, 31]. Herein, we report a timely new study on the site-specific, maxillofacial LCH lesions in 20 children covering complex manifestations, definitive diagnosis and effective treatment. Since most of our cases involve multisystem, have diverse clinical manifestations and are refractory to standard treatments, they present useful information for precision diagnosis and proper management.

## Materials and methods

### Patients

Twenty children from January 2016 to June 2021 were enrolled at our center. Inclusion criteria were as follows. (1) age of the patient at the time of treatment < 18 years old; (2) identification of LCH disease by biopsy of the lesions: positive staining of CD1a and/or CD207 (Langerin) of the lesion cells; (3) presence of maxillofacial involvement; (4) maxillofacial LCH were refractory to first-line treatment, or disease advanced quickly upon initial 1–2 weeks diagnosis (hematopoietic system and CNS involvement) requiring targeted therapy as soon as possible; (5) presence of *BRAF*^*V600E*^ or *MAP2K1* mutations, in lesion tissue or in peripheral blood plasma at diagnosis; (6) treatment with targeted therapy.

Based on the extent of disease, the patients were divided into 3 groups: single-system (SS) - the lesions in one system, multisystem (≥ two systems) without risk organs (MS - RO^−^) and multisystem with risk organs (MS - RO^+^). All patients received a complete physical examination, regular laboratory tests (complete blood count, liver function, coagulation function), imaging examinations on bone, pituitary and lung, abdominal ultrasound scanning, bone marrow aspiration. Maxillary and/or mandibular bones were examined by computed tomography (CT).

In accordance with the Declaration of Helsinki, this study was approved by the Beijing Children’s Hospital Institutional Review Board (2019-K-109) and ([2021]-E-027-Y-001), with consent from patients’ parents.

### Treatment regimen

Seventeen patients initially received chemotherapy with or without surgery, but the response was refractory, so to receive targeted therapy. The other 3 patients skipped chemotherapy treatment due to rapid disease advancement (hematopoietic system and CNS involvement) upon initial 1–2 weeks’ assessment, and immediately received targeted therapy after genetic results.

For first-line treatment, before September 2019, 6 patients were treated with prednisone and vindesine according to the BCH-2014 regimen [32] (ChiCTR2000030457); from September 2019 to April 2021, the other 11 patients were treated with prednisone and vincristine, vindesine or vinblastine according to the CCHG LCH-2019 regimen (ChiCTR1900025783) [33]. For second-line treatment, 6 of the above 17 patients were treated with cytarabine, with or without cladribine, according to CCHG-LCH-2019, because the disease response was active disease - worse (AD-W), or AD-intermediate (AD-I) at weeks 6 and 12 of first-line chemotherapy, or because LCH lesions were worsened during maintenance chemotherapy or after drug withdrawal. In short, for the seventeen patients after weeks 6 and 12 of (1st or 2nd) chemotherapy, the response was evaluated as AD-W or AD-stable (AD-S). Since they did not achieve the non-active disease (NAD) or AD-better (AD-B), they were considered refractory to standard treatments.

Targeted therapy was administered one week after the discontinuation of chemotherapy, or as soon assessment was completed due to severe disease. *BRAF*^*V600E*^ and *MAP2K1* mutations were treated with dabrafenib and trametinib, respectively. According to the previous study on dabrafenib in LCH patients [21], the dosage of dabrafenib was 2 mg/kg, orally once every 12 h (maximum dose was 150 mg per time), and the duration was 0.5 to 1.5 years. The indications for target drug discontinuation are as follows: NAD and mutation negativity in peripheral blood plasma, severe side effects, and continuous mutation positivity in peripheral blood plasma. The dose of trametinib was 0.025 mg/kg, orally once daily (maximum dose of 2 mg per day), and the duration was 0.5 to 1.5 years. The exact dose and duration would be adjusted at 1 month, 3 months after initiation of targeted therapy, and every 3 months thereafter during follow-up.

### Histopathology and immunohistochemistry study

Lesion tissues were collected from bone (6 cases), skin (8 cases), nasal soft tissue (2 cases), ocular soft tissue (2 cases), and lymph node (2 cases). Of the 6 bone samples, 4 were from the mandible, 1 from the ilium, and 1 from the femur. Immunohistochemistry (IHC) was performed to determine the expression of CD1a, Langerin, S100, Ki-67. Histiocytes in the 20 patients were consistently positive for CD1a, Langerin, and S100. In the 17 patients, Ki-67 expression was analyzed, and was defined as negative (0–5% of positive cells), weakly positive (between 5 and 50% of positive cells), and strongly positive (> 50% of positive cells) [34].

### Determination of *BRAF*^*V600E*^ and *MAP2K1* mutations

The measurements have been conducted in the same as in reference 21. Genomic DNA was extracted from 10 × 5 μm sections of formalin-fixed paraffin-embedded (FFPE) tissue using the QIAamp DNA FFPE Tissue Kit (Qiagen, Hilden, Germany) according to the manufacturer’s protocol. Next-generation sequencing was used to detect mutations in the *BRAF* and *MAPK* genes, using the HiSeq 4000 sequencing platform before 2018 and the Illumina NovaSeq 6000 sequencing platform after 2018.

After sequencing, the raw data were saved as a FASTQ format. Both Illumina sequencing adapters and low quality reads (< 80 bp) were filtered by cutadaptor software (https://cutadapt.readthedocs.io/en/stable/). The clean reads were mapped to the UCSC hg19 human reference genome using BWA software (http://bio-bwa.sourceforge.net/). The duplicated reads were removed using picard tools (http://broadinstitute.github.io/picard/), and the mapped reads were used for the detection of variation. The variants of SNP and InDel were detected by HaplotypeCaller of GATK software (https://software.broadinstitute.org/gatk/), and filtered by VariantFiltration of GATK software, following these criterions: (a) variants with mapping qualities should be < 30; (b) the total mapping quality zero reads should be < 4; (c) approximate read depth should be < 5; (d) QUAL should be < 50.0; (e) phred-scaled p-value using Fisher’s exact test to detect strand bias should be > 10.0. Then, the data would be transformed to VCF format. Variants were further annotated by ANNOVAR software (http://annovar.openbioinformatics.org/en/latest/), and associated with multiple databases, such as, 1000 genome, ESP6500, dbSNP, EXAC, Inhouse (MyGenostics), HGMD, and also predicted by SIFT, PolyPhen-2, MutationTaster, GERP++. Four steps were used to select the potential pathogenic mutations in downstream analysis: (i) Mutation reads should be more than 5, and mutation ration should be no less than 1%; (ii) The mutations should be removed, when the frequency of mutation was more than 5% in 1000 g, ESP6500, GnomAD, and Inhouse database; (iii) The mutations should be dropped, if they were in InNormal database (MyGenostics); (iV) The synonymous mutations should be removed, when they were not in the HGMD database. After that, the rest mutations should be the potential pathogenic mutations for further analysis. Plasma cell-free DNA (cfDNA) from all patients was isolated using the QIAamp Circulating Nucleic Acid Kit (Qiagen). The presence of the *BRAF*^*V600E*^ mutation was then determined using a ddPCR assay with a QX200TM Droplet Digital PCR system (Bio-Rad, Hercules, CA). Targeted sequencing of the entire coding regions of 162 (panel I) or 229 (panel II) genes of interest was performed using a customized gene panel. Detection of *BRAF*^*V600E*^ was performed by ddPCR assay with a lower limit of detection of 0.04% and a lower limit of quantitation of 0.1%.

### Follow-up and disease state assessment after targeted therapy

To review the status of maxillofacial LCH lesions after targeted therapy, the osteolytic changes of maxillary and mandibular bones were analyzed by CT/panoramic radiography, lymph nodes and soft tissue lesions by B-ultrasound/magnetic resonance imaging/18F-fluorodeoxyglucose positron emission tomography. Response to targeted therapy was assessed at 1 month, 3 months, and every 3 months thereafter.

Disease state was evaluated in terms of the maxillofacial region, and the disease response to treatment was evaluated in terms of the systemic body. The disease state at the last follow-up was categorized as complete remission (CR) and partial regression (PR) [35]. According to the criteria of the Histiocyte Society [36] and the Euro Histio Network [16], disease response is categorized as NAD, AD-B, AD-S and AD - mixed (AD-M); both are also called AD-I, plus AD-W. NAD or AD-B indicates that the LCH is responding to therapy [27]. The percentage of patients with AD-B defines the objective response rate (ORR), and the percentage of patients with AD-B and AD-S defines the disease control rate (DCR).

Adverse events included disease progression and reactivation (relapse). Disease progression is defined as progression of the original lesions or emergence of new lesion(s). Reactivation/relapse is defined as the recurrence of signs and symptoms of the active disease after complete disease resolution or a period of disease control lasting for > 3 months on maintenance therapy [17]. Adverse events were graded according to the National Cancer Institute Common Terminology Criteria for Adverse Events, v. 5.0 [37].

### Statistical analysis

Statistical analysis was performed using SPSS 26.0. Numerical data were expressed in terms of case numbers and percentages. Measurement data with a normal distribution were expressed as the mean ± standard deviation and compared by t-test, and those with a non-normal distribution were expressed as the median (P25, P75) and compared by Wilcoxon and Mann-Whitney U tests. *P* < 0.05 indicated statistical significance.

## Results

### Clinical features of patients at diagnosis

Table 1 summarizes the clinical characteristics of the study population. There are 9 males (45%) and 11 females (55%), and the median age at diagnosis was 0.92 (0.57, 1.45) years. The SS group contains 3 patients (15%), the MS-RO^−^ group contains 8 patients (40%), and the MS-RO^+^ group contains 9 patients (45%). Thus, 15% of patients with a single-system or single organ disease, and 85% with multisystem LCH.

**Table 1:** Clinical characteristics of the 20 LCH children with maxillofacial manifestations

Characteristics	*n* (%)
Gender	
Male	9 (45)
Female	11 (55)
Median age at disease onset	0.92 years
Clinical classification	
MS-RO^+^	9 (45)
MS-RO^−^	8 (40)
SS	3 (15)
Involved sites in the maxillofacial area	
Temporal bone	12 (60)
Sphenoid bone	8 (40)
Zygomatic bone	2 (10)
Maxilla	
Maxillary bone	7 (35)
Alveolar bone	3 (15)
Mandible	
Mandibular body	9 (45)
Mandibular ramus	5 (25)
Condylar	3 (15)
Soft-tissue lesions	
lymph nodes	9 (45)
Gingiva	5 (25)
Oral mucosa	4 (20)
Parotid gland	1 (5)
Other contracted sites	
Skin	10 (50)
Liver	8 (40)
Hematopoietic system/bone marrow	5 (25)
Pituitary gland	5 (25)
Lung	5 (25)
Spleen	4 (20)
Diabetes insipidus	2 (10)
CNS	1 (5)
Maxillofacial symptoms	
Swelling or masses	13 (65)
Gingivitis or oral ulceration	5 (25)
Tooth displacement/ loss/ pain	2 (10)

Lesions were observed in multiple sites in the maxillofacial region, often affecting more than one location per patient, including temporal/sphenoid/zygomatic bones, maxilla (maxillary, alveolar bones), and mandible. The most frequently involved site was the mandibular bone in 17 patients (85%), followed by the temporal bone in 12 patients (60%), the maxilla in 10 patients (50%), the sphenoid bone in 8 patients (40%), and the zygomatic bone in 2 patients (10%). The sites of contraction also extended to areas, such as skin (50%), liver (40%), bone marrow (25%), pituitary gland (25%), lung (25%), and spleen (20%). In addition, LCH lesions have invaded adjacent soft tissues, such as the lymph nodes (45%), gingiva (25%), and oral mucosa (20%). Oral symptoms include swelling or mass (65%), gingivitis or oral ulceration (25%), and tooth displacement (10%), loss, and pain.

### Outcome of targeted therapy

Table 2 details the disease extent categories, treatment regimens and outcomes. The median age at initiation of targeted therapy was 1.70 (0.93, 3.08) years. The median time of targeted therapy was 18.00 (15.25, 18.00) months. The mean follow-up of targeted therapy was 22.42 ± 2.05 months. Before targeted therapy, 16 patients were classified as AD-W and 1 patient as AD-S. At the last follow-up, 13 patients turned to AD-B and 7 patients turned to AD-S. Thus, the ORR was 65% and the DCR was 100%, indicating the significant efficacy of targeted therapy. Specifically, in 16 patients treated with dabrafenib, 12 patients (75%) were evaluated as AD-B, and the remaining 4 patients (25%) were evaluated as AD-S; the ORR was 75% and the DCR was 100%. In 4 patients treated with trametinib, 1 patient (25%) was evaluated as AD-B, and the other 3 patients (75%) were evaluated as AD-S; the ORR was 25% and the DCR was 100%.

**Table 2:** Treatment regimen and outcomes in 20 patients

No.	Disease extent categories	Treatment before TT	TT: m	Age at TT initiation: y	Disease response before TT	Disease response after TT*	Disease state after TT ^&^	Adverse events	Follow-up after TT: m
1	MS-RO^-^	1^st^ + 2^nd^ lines	D: 4.0	3.3	AD-W	AD-B	CR	P	50.0
2	MS-RO^-^	1^st^ line	D: 18.0	4.7	AD-W	AD-B	CR	-	19.5
3	MS-RO^-^	1^st^ + 2^nd^ lines	D: 18.0	3.5	AD-W	AD-S	CR	-	32.6
4	SS	Curettage + 1^st^ + 2^nd^ lines	D: 18.0	2.4	AD-W	AD-S	CR	R	34.0
5	SS	Biopsy + 1^st^ + 2^nd^ lines	D: 18.0	2.3	AD-W	AD-B	CR	-	25.6
6	MS-RO^+^	1^st^ + 2^nd^ lines	T: 18.0	0.8	AD-W	AD-S	CR	-	34.2
7	MS-RO^+^	1^st^ line	T: 24.0	0.8	AD-W	AD-S	CR	R	30.0
8	SS	Biopsy + 1^st^ line	D: 18.0	2.3	AD-W	AD-B	CR	-	24.1
9	MS-RO^-^	-	D: 18.0	2.2		AD-B	CR	P	28.0
10	MS-RO^+^	-	D: 18.0	1		AD-B	CR	P	28.0
11	MS-RO^+^	1^st^ + 2^nd^ lines	D: 11.7	3.3	AD-W	AD-B	CR	-	11.7
12	MS-RO^-^	1^st^ line	T: 18.0	1.1	AD-W	AD-S	PR	-	23.8
13	MS-RO^-^	1^st^ line	D: 18.0	0.9	AD-W	AD-S	PR	-	23.6
14	MS-RO^-^	Biopsy + 1^st^ line	D: 18.0	6	AD-W	AD-S	CR	-	21.7
15	MS-RO^+^	-	D: 18.0	1		AD-B	CR	-	21.7
16	MS-RO^+^	1^st^ line	D: 18.0	1.9	AD-W	AD-B	CR	-	21.2
17	MS-RO^+^	1^st^ line	D: 15.0	1.5	AD-S	AD-B	CR	-	19.3
18	MS-RO^+^	1^st^ line	D: 16.0	1.3	AD-W	AD-B	CR	-	16.0
19	MS-RO^-^	1^st^ line	T: 12.1	0.8	AD-W	AD-B	CR	-	12.1
20	MS-RO^+^	1^st^ line	D: 11.2	0.7	AD-W	AD-B	PR	-	11.2

TT: targeted therapy with duration time (months); 1st line: LCH-III regimen; 2nd line: CCHGLCH- 2019 regimen; D: Dabrafenib; T: Trametinib; AD-B: AD-better; AD-S: AD-stable; AD-W: AD-worse; CR: complete remission; PR: partial regression. Adverse events: progression (P), reactivation (R)

*Disease response after targeted therapy was evaluated at the last follow-up

^&^Disease state after targeted therapy was evaluated in the maxillofacial lesion at the last follow-up

In terms of disease state, at the last follow-up, seventeen patients (85%) were evaluated as CR and the remaining 3 patients (15%) were evaluated as PR. In all the 17 patients evaluated as CR, bone regeneration and intact dentition were observed. Note that the 4 patients who initially underwent surgical procedures (1 curettage and 3 biopsies), targeted therapy achieved the CR.

### Adverse events of targeted therapy

No serious adverse effects, such as squamous cell carcinoma, keratocanthoma, or primary malignant tumor, were observed after targeted therapy. The most common one was skin rash in 10 patients (50%), followed by the fever (Grade 1) in 2 patients (10%) and ulcer in 1 patient (5%). Specifically, in the 16 patients treated with dabrafenib, 6 patients (37.5%) had skin rash and 2 patients (12.5%) had fever. In the 4 patients treated with trametinib, all the patients (100%) had skin rash, and 1 patient (25%) had an ulcer.

### Activation and progression after targeted therapy

Table 3 outlines the management of five maxillofacial LCH patients who experienced progression or reactivation. In Case 1, progression occurred with a new lesion in occipital bone at the 4th month of dabrafenib treatment. The *cfBRAF*^*V600E*^ level decreased from 0.08% to 0.05%. The patient received the second-line treatment (Ara-c + VD). The other 3 patients experienced disease progression and had an increased level of *cfBRAF*^*V600E*^ after 12 months of targeted therapy. In Case 4, the *cfBRAF*^*V600E*^ level increased to 0.48% at the 20.9th month, which was 3 months later after stopping dabrafenib treatment. This patient was first evaluated 3 months later after starting dabrafenib treatment, which was AD-B (*cfBRAF*^*V600E*^ negative), and continued to receive dabrafenib treatment for 15 months without re-examination due to the Covid-19 epidemic. The oral mass completely disappeared at the last follow-up, but the bilateral orbital mass had not yet recovered, and then the patient received further chemotherapy (Ara-c + VD). In Case 9, as treated directly with dabrafenib due to diabetes insipidus, the *cfBRAF*^*V600E*^ level increased to 0.52% at the 15th month. Then, the patient received further treatment (dabrafenib + Ara-c + VD), and the *cfBRAF*^*V600E*^ level decreased to 0.02% at the last follow-up (the total duration time is 28 months). In Case 10, also treated directly with dabrafenib alone due to hemophagocytic lymphohistiocytosis (HLH), the *cfBRAF*^*V600E*^ level increased to 1.00% at the 13th month; the patient then continued to receive the treatment (dabrafenib + Ara-c + VD). Case 7 with HLH was AD-W after 1 week of first-line chemotherapy. During the first 12 months trametinib treatment, the rashes on the face gradually worsened, spreading to the trunk and limbs. Due to the severe rashes, the dosage of trametinib was reduced to 1/8 of the original dose. However, the symptom relief was not obvious, and the trametinib treatment was stopped for two weeks. Then, the dosage decreased to 1/16 of the original dose and continued for 12 months. One and a half months after stopping trametinib treatment, the lesion re-occurred in the forehead. After then, this patient was treated with a second-line chemotherapy (2CDA + Ara-c + VD) and remains under observation.

**Table 3:** Management details for 5 patients experiencing progression or reactivation

No.	TT	Adverse events after TT	Time of adverse events	cfBRAF^V600E^ 1st month to adverse	Management after adverse events
1	D	Progression	After 4 months of D	0.08% to 0.05%	Ara-c + VD
4	D	Reactivation	After 18 months of D + 3 months observation	0.00% to 0.48%	Ara-c + VD
7	T	Reactivation	After 24 months of T + 1 month observation	-	2CDA + Ara-c + VD
9	D	Progression	After 15 months of D	0.00% to 0.52%	D + Ara-c + VD
10	D	Progression	After the 13 months of D	0.45% to 1.00%	D + Ara-c + VD

2CDA: 2-Chlorodeoxyadenosine; Ara-c: aracytine; VD: Vindesine + Dexamethasone

## Discussion

**Fig. 1:**
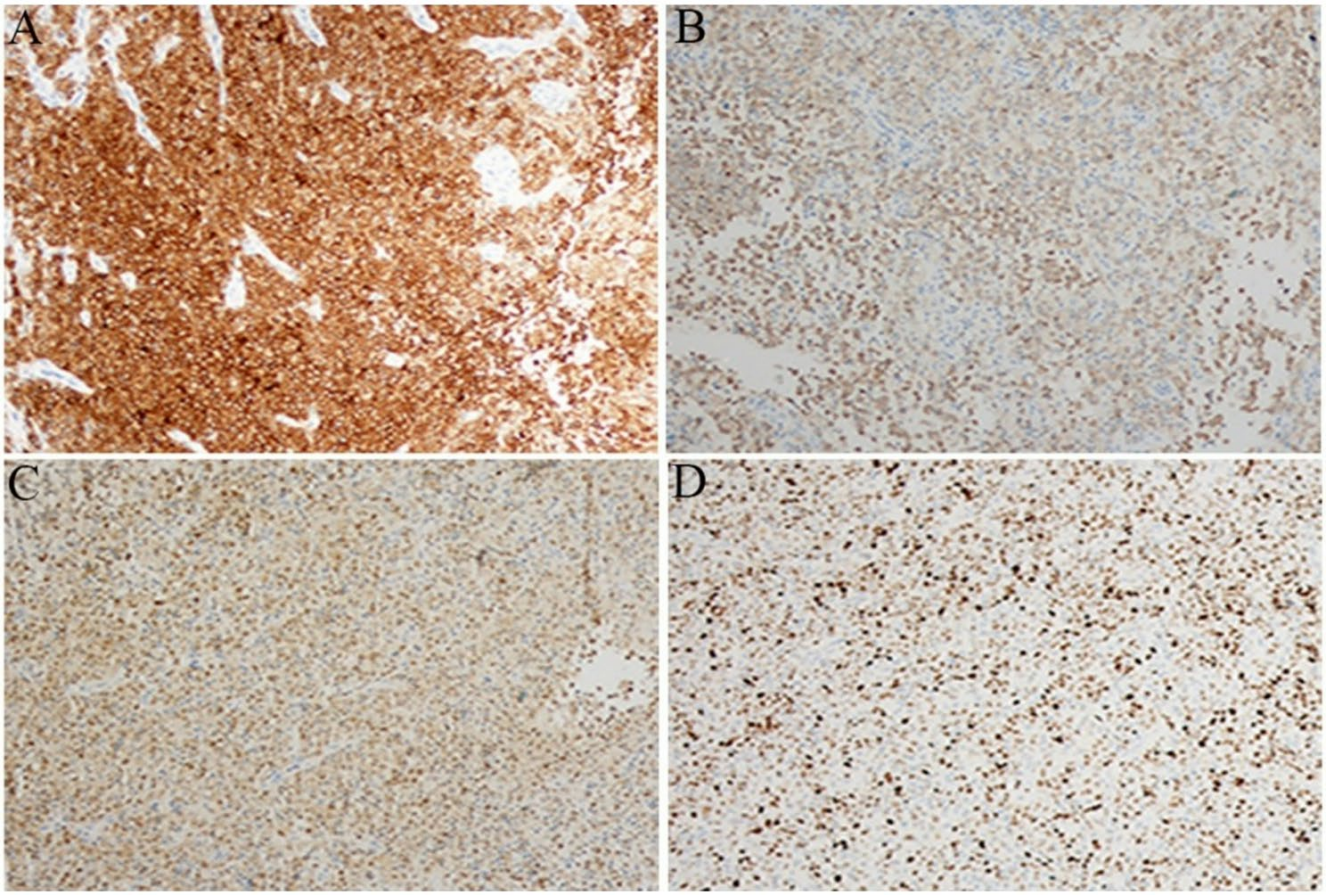
LCH lesions in case 5 (1 year female) with mandibular involvement, as diagnosed by biopsy. **A**-**D** images: immunohistochemistry positivity for CD1a, Langerin, S-100, and Ki-67, respectively. All images are at ×200 magnification of origin

Oral lesions are important as they may be the initial manifestation of the LCH or a sign of LCH reactivation [4, 10, 15], occurring in 7–10% of LCH cases [38]. In our study on the maxillofacial region, the clinical features among the 20 patients are more complicated: they include single and multiple systems, risk organ, maxilla involvement, mandibular involvement, as well as CNS-risk lesions related temporal bone, maxillary and oral involvements. The presence of the high-risk clinical manifestations, especially characterized by multiple system disease and involvement of the liver, bone marrow, spleen, has been associated with circulating cells with *BRAF*^*V600E*^ mutation [29]. This association is accompanied by a twofold increase in the risk of recurrence [29]. Children with refractory LCH frequently harbor *BRAF* mutation and tend to fail to respond to conventional chemotherapy, as seen in our 17 cases, highlighting the need for precise diagnostic approaches. Immunohistochemical staining of lesion biopsy specimens shows that histiocytes were all positive for CD1a, Langerin, S-100, and Ki-67 in all 20 patients, as illustrated in Fig. 1 (A-D) for Case 5. For the *BRAF*^*V600E*^ mutation in 16 patients and the *MAP2K1* mutation in 4 patients, tissue samples were analyzed by next-generation genomics sequencing, ensuring accurate diagnosis of refractory LCH.

Conventional chemotherapy with cytarabine and cladribine improves the five-year overall survival rate to 85% but with 100% severe toxicity and an 8% treatment-related mortality rate [39]. In contrast, the use of inhibitors targeting the *MAPK* pathway, such as vemurafenib/dabrafenib (*BRAF* inhibitor) and trametinib (*MEK1/2* inhibitor), has showed improved efficacy with mild and manageable side effects [40]. In our study, the ORR was 65% and the DCR was 100%; 85% patients achieved CR and 15% patients achieved PR, with mild adverse events - skin rash in 50% patients, followed by the fever (Grade 1) in 10% patients. So, the targeted therapy is effective.

**Fig. 2:**
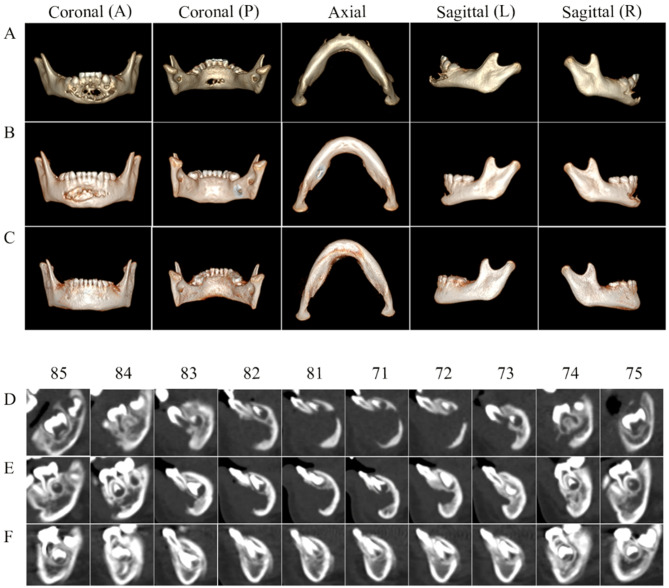
CT images of case 5 for jaw status and deciduous dentition in the mandibular. **A**: CT images of the osteolytic lesions in the mandibular at diagnosis from left to right - coronal (A-anterior, P-posterior), axial, and sagittal (L-left, R-right) scans. **B**: CT images of slow regeneration, which are 15 months later after first line treatment. **C**: CT images of fast regeneration and good jaw status, which are after 12 months of dabrafenib treatment. **D**-**F**: Sagittal CT images at different deciduous tooth sites from 85 to 75; the time acquiring the images corresponds to **A**-**C**, respectively

To treat the maxillofacial LCH lesions, surgical procedures can be used for biopsy and curettage, but complete excision is not required. Saeed et al. have reported that surgical curettage of lesion in the jaw may cause damage to the developing teeth and bone in young children [41]. In our cases, extensive or multisystem-affected lesions in the infants could not be completely removed by surgery. Since the patients in our study were infants (the median age was 0.92 years), it is important to examine the dental development and oral cavity from biopsy/curettage on the 4 patients after targeted therapy. In fact, they all had bone regeneration in the mandibular region, as shown in Fig. 2 (A-F) for Case 5. First, the CT images in Fig. 2A show the osteolytic lesions at diagnosis. After first-line chemotherapy, the CT images in Fig. 2B show only slow bone regeneration. After dabrafenib, the CT images in Fig. 2C show rapid bone regeneration. Second, the sagittal CT images in Fig. 2D at the pre-treatment and Fig. 2E at post-chemotherapy show extensive lesions involving the lower anterior teeth, as well as the corresponding permanent teeth germs. After dabrafenib, Fig. 2F reveals the timely development of both the deciduous teeth and permanent teeth germs. Note that after 5 years later, the mandibular anterior permanent teeth in the lesion region had erupted, with the corresponding deciduous teeth exfoliated, which suggests normal development of permanent teeth. (See supporting information).

In terms of a specific type of mutation, a prior study shows that 26 out of 36 patients (72.2%) with lesions in the head and neck were identified with *BRAF*^V600E^ mutation [42]. Our data are similar: 80% had the *BRAF*^*V600E*^ mutation, and 20% had the *MAP2K1* mutation. Furthermore, past studies indicated that a high level of *cfBRAF*^*V600E*^ in refractory LCH with risk organ involvement [17] was associated with high-risk features and intolerance to chemotherapy [21, 43], and the persistently positive *cfBRAF*^*V600E*^ was associated with a high risk of reactivation after drug discontinuation [43, 44].

**Fig. 3:**
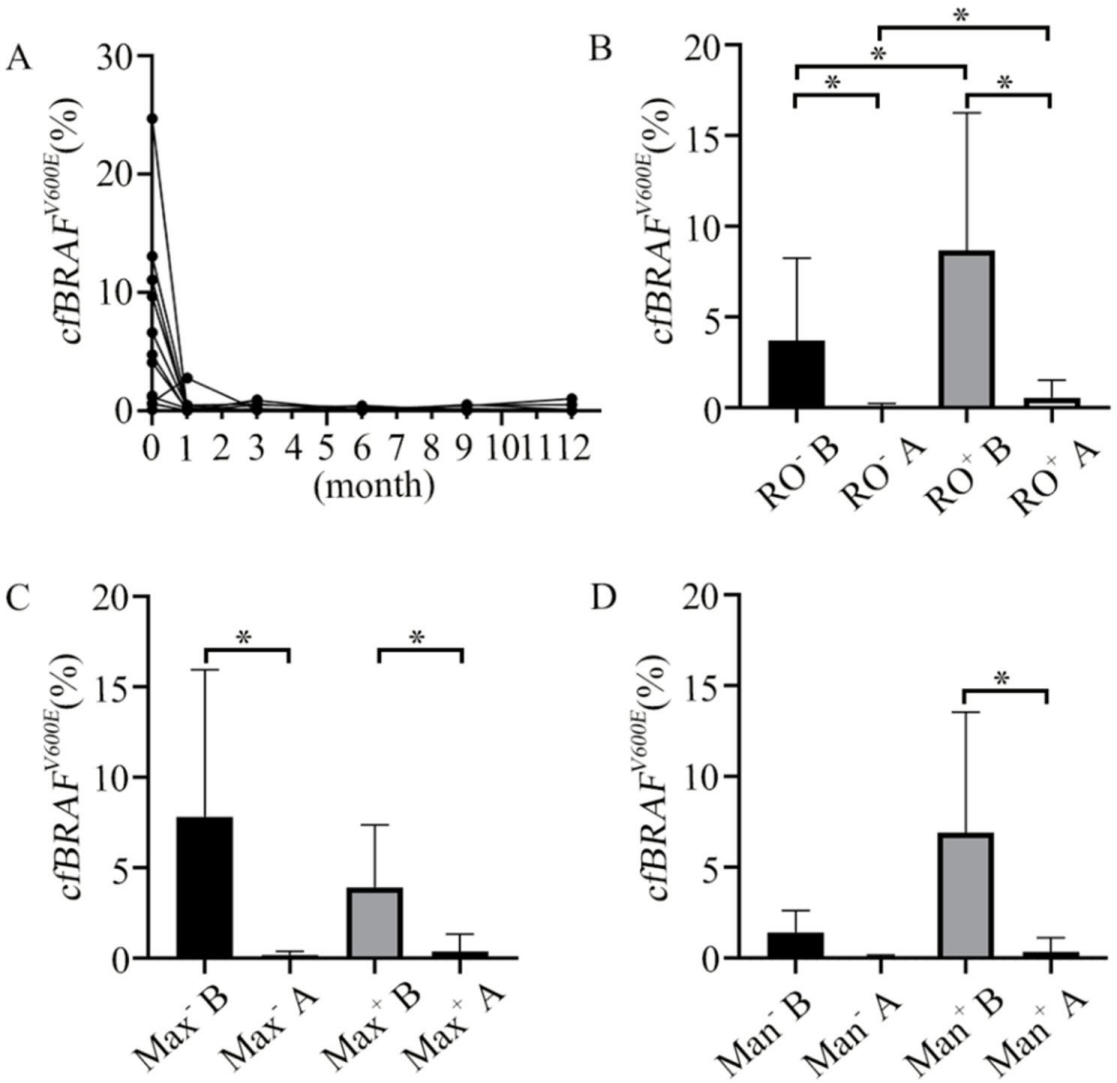
*cfBRAF*^*V600E*^ level among the 16 patients treated with dabrafenib. **A**: *cfBRAF*^*V600E*^ level in each patient decreased significantly after the first month treatment, and remained negative at the 12th month. **B**-**D**: Comparison of *cfBRAF*^*V600E*^ level before vs. at first month dabrafenib treatment (**B** vs. **A**) of being with vs. without RO groups (RO^+^ vs. RO^−^), maxilla groups (Max^+^ vs. Max^−^), and mandibular groups (Man^+^ vs. Man^−^). *: *P* < 0.05, the bars represent the standard deviation

Moreover, in Fig. 3A, after dabrafenib treatment in the 16 patients with *cfBRAF*^*V600E*^ mutation, the *cfBRAF*^*V600E*^ levels in peripheral blood decreased sharply at the end of the first month and remained low throughout the 12 months. Wilcoxon paired t-test shows that the *cfBRAF*^*V600E*^ levels were lower at the end of the first month, compared to before the targeted treatment (*P* < 0.001). To analyze the location-specific change in the *cfBRAF*^*V600E*^ level, we divided the 16 patients into two sub-groups, e.g. RO involvement before/after targeted treatment (RO^+^ B vs. RO^+^ A) or no RO involvement before/after targeted treatment (RO^−^ B vs. RO^−^ A), maxilla involvement before/after targeted treatment (Max^+^ B vs. Max^+^ A), and mandibular involvement before/after targeted treatment (Man^+^ B vs. Man^+^ A). The sub-group analysis shows that, as in Fig. 3 (B-D), the decrease in *cfBRAF*^*V600E*^ level was all significant in the RO^+^ (*P* = 0.02) and RO^−^ (*P* = 0.02) sub-groups, in the Max^+^ (*P* = 0.02) and Max^−^ (*P* = 0.02), and the Man^+^ (*P*<0.001) sub-groups. So, the rapid decrease in the *cfBRAF*^*V600E*^ level at the first month of treatment is independent of the disease sites. Note that there were only 2 patients in the Man^−^ sub-group, so the statistical comparison was not performed. Furthermore, the Mann-Whitney U test shows that there was a significantly higher level of *cfBRAF*^*V600E*^ in RO^+^ than in RO^−^, either before (*P* = 0.03) or after 1 month (*P* = 0.03) of dabrafenib treatment, indicating that the risk organ involvement tends to be more associated with the level of *cfBRAF*^V600E^. In contrast, there was no significant difference in *cfBRAF*^*V600E*^ between Max^+^ and Max^−^, either before dabrafenib treatment (*P* = 0.80) or after the first month of dabrafenib treatment (*P* = 0.50). This may further highlight the important relationship between RO involvement and the level of *cfBRAF*^*V600E*^.

**Fig. 4:**
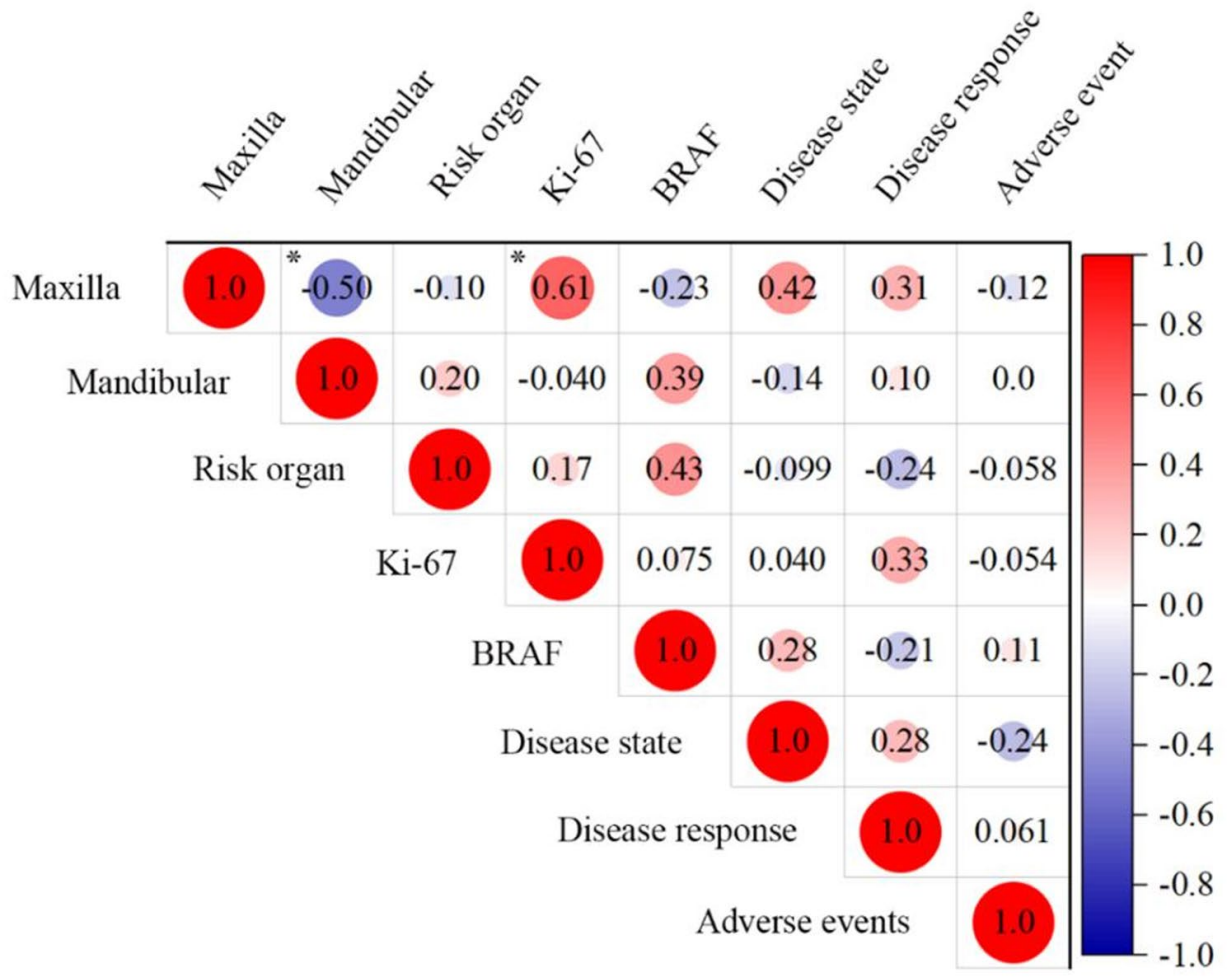
Correlation among clinical features, laboratory indices and treatment outcome, as analyzed by the Kendall correlation coefficient. *: *P* < 0.05. *cfBRAF*^*V600E*^ mutation is from the 16 patients, and Ki-67 expression in FFPE tissue samples is from the 17 patients. *BRAF*: the level of *cfBRAF*^*V600E*^ before target treatment. Disease state: disease state of maxillofacial lesion at the last follow-up. Disease response: disease response of target therapy at the last follow-up

In addition, Fig. 4 shows the Kendall’s correlation coefficient among the maxillary, mandibular, and risk organ involvements, Ki-67 and *cfBRAF*^*V600E*^ levels before targeted therapy, the disease state of maxillofacial lesion, disease response and adverse events. There are positive correlations between Ki-67 expression and maxillary involvement (*r* = 0.61; *P* < 0.05), and negative correlation between maxillary and mandibular involvements (*r* = -0.50; *P* < 0.05). Also, maxilla involvement tends to result in a better disease state (*r* = 0.42; *P* = 0.07), and risk organ involvement tends to have a higher level of *cfBRAF*^*V600E*^ before dabrafenib treatment (*r* = 0.43; *P* = 0.05). In our cohort, Ki-67 > 50% appeared in 5 patients and maxilla LCH appeared in 10 patients, and their correlation is positive and statistically significant. This association implies that the LCH cells and surrounding cells in maxilla lesions may have high proliferative activity. A larger sample size in future will confirm if this is the case.

This study is limited by its small sample size and relatively short follow-up time, which precludes the event free Kaplan-Meier survival analysis. In the future, *cfBRAF*^*V600E*^ or *BRAF*^*V600E*^ in peripheral blood mononuclear cells should be tried to predict the disease progression/reactivation/treatment response. Further assays targeting other molecular abnormalities including *MAP2K1* mutations should also be established.

## Conclusion

In this report, 20 LCH children with maxillofacial manifestations are studied in terms of diverse clinical features, careful laboratory diagnoses, and proper management. In particular, 17 out of the 20 patients are refractory to standard treatments. Targeted therapy demonstrates better efficacy and tolerability compared to surgery or chemotherapy. We also found the positive correlation of the Ki-67 expression with the maxillary involvement, as well as the positive correlation of the risk organ involvement with the level of *BRAF*^*V600E*^. This study will provide valuable information for this type of rare maxillofacial LCH with *BRAF*^*V600E*^ / *MAP2K1* mutations.

## Supplementary Information

Below is the link to the electronic supplementary material.

Supplementary Material 1

## Data Availability

Available upon request.

## Code availability

Available upon request.

## Abbreviations

LCH: Langerhans cell histiocytosis
CNS: Central nervous system
BRAF: V-Raf murine sarcoma viral homolog B1
MAPK: Mitogen-activated protein kinase
MAP2K1/MEK1: Mitogen-activated protein kinase 1
SS: Single system
MS - RO^−^: Multisystem (≥ two systems) without risk organs
MS - RO^+^: Multisystem with risk organs
CT: Computed tomography
AD-W: Active disease - worse
AD-I: Active disease - intermediate
AD-S: Active disease – stable
AD-B: Active disease – better
AD-M: Active disease – mixed
NAD: Non-active disease
FFPE: Formalin-fixed paraffin-embedded
cfDNA: Cell-free DNA
ORR: Objective response rate
DCR: Disease control rate
CR: Complete remission
PR: Partial regression
IHC: Immunohistochemistry
TT: Targeted therapy
1st line: LCH-III regimen
2nd line: CCHGLCH- 2019 regimen
D: Dabrafenib
T: Trametinib
P: Progression
R: Reactivation
2CDA: 2-Chlorodeoxyadenosine
Ara-c: Aracytine
VD: Vindesine + Dexamethasone
