# Supplementary material for: Targeted therapy for Langerhans cell histiocytosis with maxillofacial involvement in 20 children
Source: Orphanet J Rare Dis. 2026 Mar 26;21:109. doi: 10.1186/s13023-026-04301-w (PMC13023140; doi:10.1186/s13023-026-04301-w)
Supplement: Supplementary file 1 — Supplementary Material 1 [file 13023_2026_4301_MOESM1_ESM.docx]

**Targeted therapy for Langerhans cell histiocytosis with maxillofacial involvement in 20 children**

Yang Jiang^*^, MD, PhD ^a#^, Zhigang Li, PhD ^b#^, Ying Yang, MD, PhD ^b^, Rui Zhang, MD ^b*^, Tianyou Wang, MD ^b*^, Guoxia Yu, DDS, PhD ^a*^

a: Department of Stomatology, Beijing Children’s Hospital, Capital Medical University, National Center for Children’s Health (NCCH), Beijing, China, 100045.

b: Laboratory of Hematologic Diseases, hematology Center, Beijing Pediatric Research Institute, Beijing Children's Hospital, Capital Medical University, National Center for Children’s Health, Beijing 100045, China; Beijing Key Laboratory of Pediatric Hematology Oncology, Beijing Children’s Hospital, Capital Medical University, National Center for Children’s Health, Beijing 100045, China; National Key Discipline of Pediatrics, Capital Medical University, Beijing 100045, China; Key Laboratory of Major Diseases in Children, Ministry of Education, Beijing 100045, China.

# Yang Jiang and Zhigang Li contributed equally to this work.

* **Corresponding Authors**:

Guoxia Yu, DDS, PhD; email: yuguoxia@bch.com.cn

Tianyou Wang, MD; email: [wangtianyou@bch.com.cn](mailto:wangtianyou@bch.com.cn)

Rui Zhang, MD; [ruizh1973@126.com](mailto:ruizh1973@126.com)

Yang Jiang, MD; [BJETYYKQ2016@163.COM](mailto:BJETYYKQ2016@163.COM)

**Supporting information**


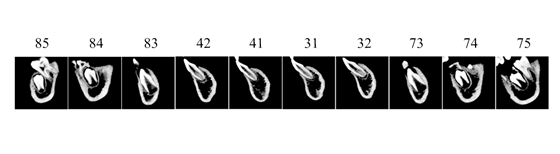


Figure 1S. Sagittal CT images of the patient (Case 5) for jaw status and mixed dentition in the mandibular, which were 5 years later (compared to them in Figure 2F).

At the recent follow-up in January 2026, Figure 1S shows the sagittal CT images for the patient (Case 5; currently 8 years old). The mandibular bone in the previously affected region was well-developed, providing adequate support for the normal eruption of the permanent teeth. Permanent teeth 31, 41, 32, and 42 in the mandibular anterior region had erupted, with the corresponding deciduous teeth exfoliated. Deciduous teeth 73-75 and 83-85 had root resorption, with the corresponding permanent tooth germs showing normal development. In addition, the mandibular first permanent molars had fully erupted.
